# Supplementary material for: Clinical Significance and Regulation of ERK5 Expression and Function in Cancer
Source: Cancers (Basel). 2022 Jan 11;14(2):348. doi: 10.3390/cancers14020348 (PMC8773716; doi:10.3390/cancers14020348)
Supplement: Supplementary file 1 [file cancers-14-00348-s001.zip › cancers-1520563-supplementary.pdf]

cancers

MDPI

Table S1. Summary statistics of the TCGA cohort of interest. Legend: BLCA, Bladder Urothelial Carcinoma; BRCA, Breast invasive carcinoma; COAD, Colon adenocarcinoma; HNSC, Head and Neck squamous cell carcinoma; LUAD, Lung adenocarcinoma; LUSC, Lung squamous cell carcinoma; OV, Ovarian serous cystadenocarcinoma; SKCM, Skin Cutaneous Melanoma; UCEC, Uterine Corpus Endometrial Carcinoma; #, relative fractions among BRCA cases.

	Molecular Subtype (BRCA)	Overall (N=3850)
**Sample type**		
Primary Tumor		3850 (100%)
**Tumor site**		
TCGA-BLCA		406 (10.5%)
TCGA-BRCA		1070 (27.8%)
	Normal	40 (3.7%#)
	LumA	550 (51.4%#)
	LumB	214 (20.0%#)
	Her2	80 (7.5%#)
	Basal	186 (17.4%#)
TCGA-COAD		273 (7.1%)
TCGA-HNSC		520 (13.5%)
TCGA-LUAD		507 (13.2%)
TCGA-LUSC		498 (12.9%)
TCGA-OV		302 (7.8%)
TCGA-SKCM		98 (2.5%)
TCGA-UCEC		176 (4.6%)
**Stage**		
Stage I		876 (22.8%)
Stage II		1336 (34.7%)
Stage III		1045 (27.1%)
Stage IV		593 (15.4%)
**ERK5 (log_2RSEM)**		
Mean (SD)		8.64 (0.619)
Median [Min, Max]		8.63 [5.15, 12.0]

Table S2. Summary statistics of the TCGA cohort of interest showing the Stage distribution of patients and ERK5 expression across tumor sites. Legend: BLCA, Bladder Urothelial Carcinoma; BRCA, Breast invasive carcinoma; COAD, Colon adenocarcinoma; HNSC, Head and Neck squamous cell carcinoma; LUAD, Lung adenocarcinoma; LUSC, Lung squamous cell carcinoma; OV, Ovarian serous cystadenocarcinoma; SKCM, Skin Cutaneous Melanoma; UCEC, Uterine Corpus Endometrial Carcinoma.

	Stage I (N=876)	Stage II (N=1336)	Stage III (N=1045)	Stage IV (N=593)	Overall (N=3850)
**Sample type**					

Primary Tumor	876 (100%)	1336 (100%)	1045 (100%)	593 (100%)	3850 (100%)
**Tumor site**					
TCGA-BLCA	2 (0.2%)	130 (9.7%)	140 (13.4%)	134 (22.6%)	406 (10.5%)
TCGA-BRCA	182 (20.8%)	619 (46.3%)	249 (23.8%)	20 (3.4%)	1070 (27.8%)
TCGA-COAD	45 (5.1%)	109 (8.2%)	80 (7.7%)	39 (6.6%)	273 (7.1%)
TCGA-HNSC	27 (3.1%)	83 (6.2%)	94 (9.0%)	316 (53.3%)	520 (13.5%)
TCGA-LUAD	275 (31.4%)	122 (9.1%)	84 (8.0%)	26 (4.4%)	507 (13.2%)
TCGA-LUSC	245 (28.0%)	162 (12.1%)	84 (8.0%)	7 (1.2%)	498 (12.9%)
TCGA-OV	1 (0.1%)	21 (1.6%)	242 (23.2%)	38 (6.4%)	302 (7.8%)
TCGA-SKCM	2 (0.2%)	66 (4.9%)	27 (2.6%)	3 (0.5%)	98 (2.5%)
TCGA-UCEC	97 (11.1%)	24 (1.8%)	45 (4.3%)	10 (1.7%)	176 (4.6%)
**ERK5 (log_2RSEM)**					
Mean (SD)	8.59 (0.591)	8.53 (0.615)	8.69 (0.629)	8.85 (0.585)	8.64 (0.619)
Median [Min, Max]	8.59 [5.15, 11.0]	8.48 [6.38, 11.2]	8.71 [6.03, 11.3]	8.90 [6.22, 12.0]	8.63 [5.15, 12.0]
